# Case of a Large Fibro-Cartilaginous Tumour Successfully Removed from the Neck

**Published:** 1839-11-30

**Authors:** Thomas Miller

**Affiliations:** Professor of Surgery in the Columbian College, D. C.; Washington City, D. C.


*Case of a large Fibro-Cartilaginous Tumour successfully removed from the Neck.* By THOMAS MILLER, M. D., *Professor of Surgery in the Columbian College, D. C.*

[Reported by S. LAWRIE, M. D.]

George West, of Fauquier county, Va., coloured, æt. sixty-four, of good constitution and habits, nineteen years since discovered a tumour on the left side of his neck, which was of slow growth until within the last ten years, during which time it increased to such a size as to become almost insupportable, and to interfere with his respiration when recumbent. The appearance of the tumour when the patient presented himself to Prof. Miller, was as follows:—Its shape somewhat that of a truncated cone. The space occupied by it was bounded by a line extending from the mastoid process obliquely over the angle of the jaw to the thyroid cartilage, downwards to within an inch of the sternal end of the clavicle, running parallel with that bone nearly to the outer border of the traperius, then ascending to the point of commencement. The mass of the tumour could be readily moved, its weight having formed a neck by which it was pendant. The skin was healthy, and not adherent. Its surface had a number of nodules, some of which were hard, others soft, and communicating to the touch the sense of fluctuation, with the exception of a large cutaneous artery at the lower portion of the tumour, no unusual vascularity was discoverable. No symptoms of malignancy were evinced, and there was freedom from pain. On the 30th of October, at the City Almshouse, Prof. Miller, aided by Dr. Hall, and Professors Sewall and May, operated as follows:—An incision was made, with the curve downwards, dividing the superficial fascia and platysma myoides from the ear to the thyroid cartilage. The flap was then dissected upwards, until the base of the tumour was reached. Before this point of the operation was gained, numerous branches of the facial artery required ligatures.

The second incision was made, encircling the lower portion of the tumour, with the curve upwards. Branches of the occipital artery were taken up, and the external jugular vein tied, to prevent the admission of air. The sterno-cleido-mastoid muscle was then dissected from the surface of the tumour on which it was stretched. The base was again reached, and the mass easily and safely removed from the fascia propria, to which it was attached by loose cellular substance. The weight of the tumour, after extirpation, was seven and a half pounds; the circumference of its base, twenty-five inches; its greatest diameter, nine inches; its least, seven. Its structure was fibro-cartilaginous, having cells which contained a dark, pulpy, and cherry matter.

*Nov. 14th.*—The patient is in good health,—has had no bad symptoms since the operation. The ligatures have come away, and the wound nearly healed.

*Washington City, D. C.*, November, 1839.

